# Revised taxonomic classification of the *Stenotrophomonas* genomes, providing new insights into the genus *Stenotrophomonas*

**DOI:** 10.3389/fmicb.2024.1488674

**Published:** 2024-12-12

**Authors:** Ze-Lin Yu, Rui-Bai Wang

**Affiliations:** National Key Laboratory of Intelligent Tracking and Forecasting for Infectious Disease, National Institute for Communicable Disease Control and Prevention, Chinese Center for Disease Control and Prevention, Beijing, China

**Keywords:** *Stenotrophomonas*, average nucleotide identity, taxonomy, species classification, genetic diversity

## Abstract

**Background:**

*Stenotrophomonas* strains are important opportunistic pathogens with great potential applications in industry and agriculture. Their significant genetic and phenotypic diversity has led to several changes in their taxonomic localization and was prone to inaccurate species classification based on traditional identification methods.

**Methods:**

All 2,615 genomes of the genus *Stenotrophomonas* were obtained from the NCBI genome database. Genomic methods, including average nucleotide identity (ANI), were used to evaluate the 31 defined species. After evaluating the ANI thresholds applicable to *Stenotrophomonas*, the species classification of all submitted genomes was revised.

**Results:**

Compared to the reference genomes of each species, 41.17% of the submitted *Stenotrophomonas* genomes had ANI values below 95, and 8.58% of the genomes were even below 90%. Moreover, 45.3% (705/1555) of the *S. maltophilia* strains actually belonged to other species within the *S. maltophilia* complex (Smc), or even to distant relatives outside the Smc. Based on the ANI threshold values of 95 and 90% for species and complexes confirmed to be applicable to *Stenotrophomonas*, 2,213 submitted *Stenotrophomonas* genomes were re-divided into 116 ANI genome species.

**Conclusion:**

The results confirmed that 16S rRNA gene sequencing has low discriminability for the closely related *Stenotrophomonas* species. The annotated species of a considerable strain were indeed incorrect, especially since many *S. maltophilia* strains did not belong to this representative pathogenic species of *Stenotrophomonas*. This makes it necessary to reconsider the evolutionary relationship, pathogenicity, and clinical significance of *Stenotrophomonas*.

## 1 Introduction

The genus *Stenotrophomonas* comes under the family *Lysobacteracea*e (*Xanthomonas* group), the order *Lysobacterales*, the class *Gammaproteobacteria*, and the phylum *Pseudomonadota* (Palleroni and Bradbury, 1993; Brooke, 2021). The members of the genus *Stenotrophomonas* are ubiquitous in diverse habitats, with soil and plants serving as their main environmental reservoirs (Ryan et al., 2009). This microorganism is associated with the plant rhizosphere, has an important role in the elemental cycling of sulfur and nitrogen, and engages in beneficial interactions with plants to promote their growth and health (An and Berg, 2018; Pérez-Pérez et al., 2020; Kumar et al., 2023). In addition, these bacteria can degrade complex compounds and pollutants and thus have a potential role in environmental remediation (Venkidusamy and Megharaj, 2016; Xiong et al., 2020). However, a few members of this genus are pathogens that can cause various infections and certainly impact human health worldwide. The representative species is *Stenotrophomonas maltophilia*, the first member of this genus and also a globally emerging multidrug-resistant and opportunistic pathogen that has attracted much attention (Brooke, 2012; Chang et al., 2015). According to global clinical data, the attributed mortality rate of bacteremia caused by this bacterium is as high as 65% (Hua et al., 2013).

Owing to its well-known ecological and phenotypic diversity and intraspecific heterogeneity (Palleroni and Bradbury, 1993), the taxonomic status of the genus *Stenotrophomonas* has undergone several revisions (Tamma et al., 2022) and was once referred to as *Pseudomonas* and *Xanthomonas* (Swings et al., 1983), as well as the composition of the so-called *S. maltophilia* group/complex (Smc) (Ochoa-Sánchez and Vinuesa, 2017; Vinuesa et al., 2018). To date, this genus has different taxonomic structures in different databases. In the National Center for Biotechnology Information (NCBI) taxonomy (https://www.ncbi.nlm.nih.gov/datasets/taxonomy/), the genus *Stenotrophomonas* currently comprises 31 classified species, 3,044 unclassified species, and 65 unclassified species from environmental samples. Smc is composed of three classified species, *S. maltophilia*, *S. sepilia,* and *Pseudomonas hibiscicola*, and 24 unclassified species. While present in Jean Euzeby’s List of Prokaryotic Names with Standing in Nomenclature (LPSN, https://www.bacterio.net/genus/stenotrophomonas), only 31 classified species are included as child taxa in the genus *Stenotrophomonas* (23 validly published under the ICNP and 21 with correct names), of which 28 species are consistent with NCBI. In the genome taxonomy database structured based on genome phylogeny (Parks et al., 2020) (GTDB, https://gtdb.ecogenomic.org/), 1,302 genomes of the genus *Stenotrophomonas* are divided into 112 species, of which 45 species have not been assigned names and many classified species are subdivided. For example, *S. maltophilia* is divided into 34 *S. maltophilia* species (A, AA, G, K, etc.) and *S. rhizophila* is divided into 4 *S. rhizophila* species; moreover, *S. panacihumi* and 3 *S.* sp. are removed from the genus *Stenotrophomonas* and placed under a new genus, *Stenotrophomonas_A* (https://gtdb.ecogenomic.org/tree?r=g__Stenotrophomonas_A).

Taxonomic methods include phenotypic, physiological, and genotypic characteristics, and the way microbiologists describe and classify species has undergone a major revision in the light of genomics (Konstantinidis and Tiedje, 2007; Sentausa and Fournier, 2013; Vandamme and Peeters, 2014). Among these methods, 16S rRNA sequencing is one of the most universal single-gene tools for differentiating bacteria, with 98.65% similarity generally set as the threshold for species differentiation (Ludwig and Klenk, 2001; Kim et al., 2014; Rosselló-Móra and Amann, 2015). However, this threshold does not apply to *Stenotrophomonas*. Gröschel MI et al. defined *S. maltophilia* strains in Smc with 16S rRNA gene sequence similarities > 99.0% (Svensson-Stadler et al., 2012; Gröschel et al., 2020), and our study also showed that the similarities between *S. pigmentata* and *S. terrae* (99.24%) as along with the *S. humi* strain (98.96%) were greater than 98.65% (Li et al., 2024). Multilocus sequence typing (MLST) was used as a standard typing tool, enabling the investigation of evolutionary relationships among species classified within the same genus (Rizek et al., 2018). However, only *S. maltophilia* has a well-established combined MLST scheme available in the PubMLST database (Kaiser et al., 2009); other species lack a standardized definition. DNA–DNA hybridization (DDH) has been used as the gold standard for circumscribing prokaryotic species at the genomic level (Goris et al., 2007; Richter and Rosselló-Móra, 2009). Due to its complexity and time-consuming nature, DDH has been gradually replaced by digital DDH (dDDH) and average nucleotide identity (ANI) (Jain et al., 2018; Meier-Kolthoff and Göker, 2019). The recommended cutoff point of 70% DDH for species delineation corresponded to 95% ANI (Gosselin et al., 2022). In addition, a study suggested that 98% ANI is the accurate threshold for distinguishing subspecies (Pearce et al., 2021), although it is not recognized as a standard cutoff value in the taxonomy of different prokaryotes.

The number of *Stenotrophomonas* species has increased rapidly in the past 10 years, and an increasing number of *Stenotrophomonas* genomes have been deposited in the NCBI database (Li et al., 2024). The correct definitions of the genus *Stenotrophomonas*, its species, and the *S. maltophilia* complex and the correct species annotation of the submitted genomes are necessary to obtain accurate bioinformatics analysis results, which are crucial for the identification of new species and pathological epidemiology research and are the basis for a correct understanding of this genus. However, the submitted genomes in different periods were species-annotated based on different levels of strain identification methods. Therefore, we compared the discrimination and operability of several taxonomic methods for *Stenotrophomonas* and revised the species classification of all submitted genomes in this study.

## 2 Materials and methods

### 2.1 *Stenotrophomonas* genome retrieval and processing

All 2,615 genomes of the genus *Stenotrophomonas* were obtained from the NCBI genome database (https://www.ncbi.nlm.nih.gov/datasets/genome/) as of 1 April 2024. Among them, 402 genomes noted as atypical assemblies (including 22 contaminated genomes, 74 too large and too small genomes, and 306 genomes from unverified sources) were excluded. A total of 2,213 genomes were ultimately included in this analysis. The genome size and GC content of the genomes were ascertained using Seqkit v2.8.2 (https://github.com/shenwei356/seqkit) (Shen et al., 2024).

### 2.2 Analysis of reference genomes based on 16S rRNA gene identity, dDDH, and ANI

Currently, there are 31 recognized species of the genus *Stenotrophomonas* in the NCBI Taxonomy database. Notably, *S. indologenes* and *S. detusculanense* do not have available genome sequences. Furthermore, neither *S. aracearum* nor *S. oahuensis* has a reference genome assigned in the NCBI database; their only available genomes served as reference genomes. Two novel species of the genus *S. pigmentate* and *S. tuberculopleuritidis* (610A2 and 704A1, currently temporarily classified as *S.* sp.) identified in our previous research (Li et al., 2024) were also incorporated as reference genomes for the study. Consequently, a total of 31 reference genomes were included in this study (Supplementary Table S1).

The 16S rRNA gene sequences were extracted from the genomes using barrnap v0.9 (https://github.com/tseemann/barrnap). Pairwise similarities between 16S rRNA genes were assessed through alignment using BLAST+ v2.15.0 (http://blast.ncbi.nlm.nih.gov/Blast.cgi). The ANI similarity matrix was constructed using skani v0.2.1(https://github.com/bluenote-1577/skani) (Shaw and Yu, 2023) and FastANI v1.33 (https://github.com/ParBLiSS/FastANI) (Jain et al., 2018). dDDH values of the reference genomes were compared using the GGDC 3.0 (https://ggdc.dsmz.de/ggdc.php) (Meier-Kolthoff et al., 2022). The visualization of the results was conducted using Matplotlib v3.8.4 (https://matplotlib.org/) and Seaborn v0.11.0 (https://seaborn.pydata.org/) in Python 3.12.1.

### 2.3 Phylogenetic analysis based on core-genome alignments

For the phylogenetic analysis of all reference genomes, *Pseudoxanthomonas broegbernensis* DSM 12573 was selected as the outgroup. We used Prokka v1.14.6 (https://github.com/tseemann/prokka) (Seemann, 2014) to annotate the genomes. The output GFF-formatted files from Prokka were used as input files for Roary v3.13.0 (https://github.com/sanger-pathogens/Roary) (Page et al., 2015) to calculate the genus *Stenotrophomonas* core and pan-genomes with the minimum blastp percentage identity set at 90%. The phylogenetic tree was constructed using FastTree 2(https://github.com/PavelTorgashov/FastTree) (Price et al., 2010) based on the multi-FASTA alignment of all of the core genes and visualized using Tree Visualization By One Table (tvBOT) (Xie et al., 2023).

### 2.4 Consistency between skani and FastANI

The ANI value matrix between the 31 reference genomes and the ANI of all the *Stenotrophomonas* genomes compared to the 31 reference genomes was generated using both FastANI and skani. For each species, genomes were categorized into three groups based on their ANI compared to the reference genome as follows: ANI ≥ 95, 95% > ANI ≥ 90%, and ANI < 90%. The difference in classification between the two ANI tools was analyzed using the McNemar–Bowker test, and the consistency was evaluated by the kappa coefficient. The difference between the ANI values calculated by the two tools after stratifying was evaluated using a paired *t*-test. All statistical analyses were conducted using R version 4.4.0 (https://cran.r-project.org/bin/windows/base/old/4.4.0/), with *p* < 0.05 indicating statistical significance.

### 2.5 ANI clustering and evaluation of ANI cutoff values

The ANI matrix for all genomes of the genus *Stenotrophomonas* was generated using skani or FastANI. A helper Python script provided using skani was employed to visualize and cluster the lower-triangular similarity matrix that skani output. Building upon this script, the fcluster function from Scipy v1.13.1 (https://docs.scipy.org/doc/scipy/) was integrated to actually obtain clusters by setting an ANI threshold.

We examined the distribution of pairwise ANI values separately between all genomes of the genus *Stenotrophomonas* (*n* = 2,213) and between all genomes of *S. maltophilia* (*n* = 1,555) using skani. In our evaluation, one genome was taken as the reference genome, and all genomes (including itself) were taken as query genomes. A total of n-squared pairwise comparisons were executed. The density curve of the pairwise ANI values was plotted using ggplot2 (https://ggplot2.tidyverse.org/) in R version 4.4.0.

The ANI cutoff value was evaluated through the analysis of clustering results and the density distribution. Based on the ANI cutoff values, genomes within the genus *Stenotrophomonas* were reclassified into the designated species.

## 3 Results

### 3.1 Composition and general characteristics of the genomes of the genus *Stenotrophomonas*

**Figure 1:**
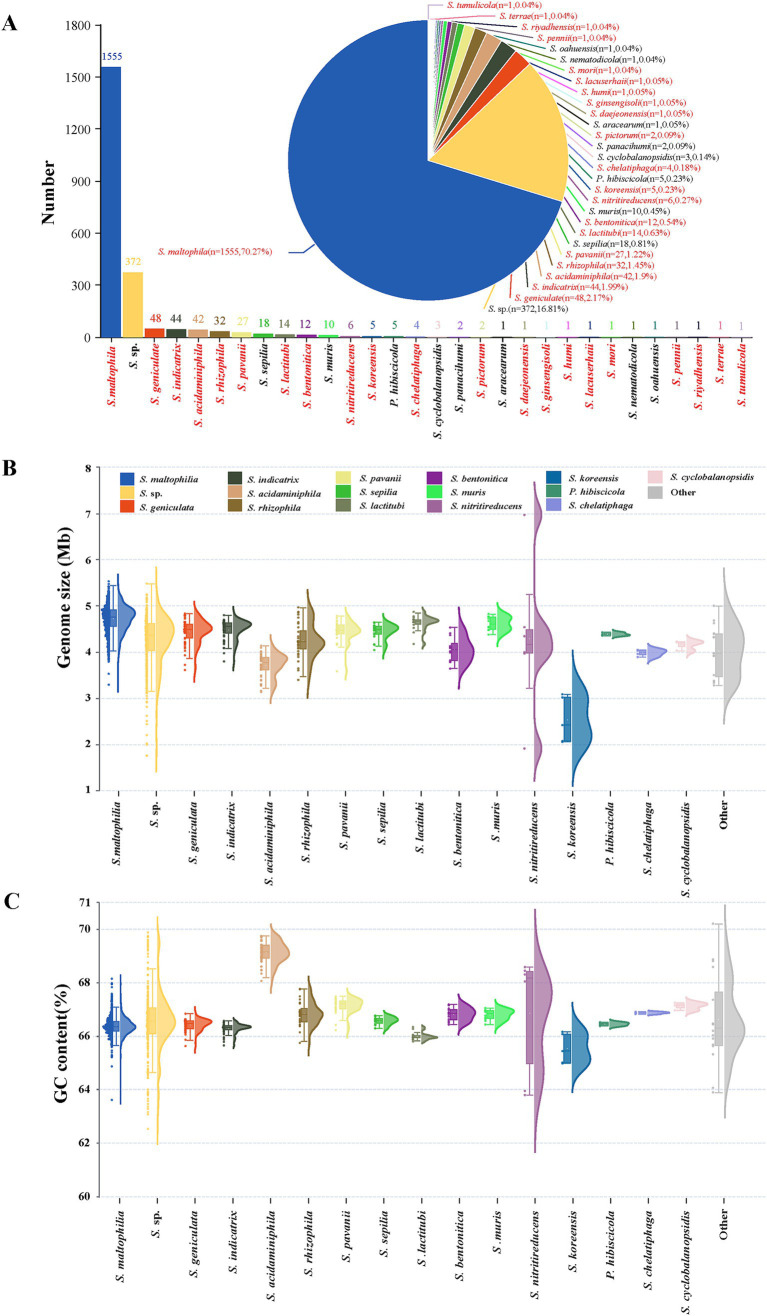
*Stenotrophomonas* species and general genomic characteristics of genomes analyzed in this study. **(A)** *Stenotrophomonas* species, arranged in descending order from left to right based on their submitted genome numbers in the NCBI genome database. In total, 21 species with validly published and correct names are marked in red. **(B,C)** show the genome size and GC content of each *Stenotrophomonas* species using raincloud plots, respectively. The left beeswarm plots depict the distribution of individual data points with ‘raindrops’, the central box plots indicate the median, interquartile range, and potential outliers, and the right kernel density plots illustrate the probability density function of the continuous variable. Other refers to species with only one or two genomes, including *S. aracearum* (*n* = 1), *S. daejeonensis* (*n* = 1), *S. ginsengisoli* (*n* = 1), *S. humi* (*n* = 1), *S. lacuserhaii* (*n* = 1), *S. mori* (*n* = 1), *S. nematodicola* (*n* = 1), *S. panacihumi* (*n* = 2), *S. pennii* (*n* = 1), *S. pictorum* (*n* = 2), *S. riyadhensis* (*n* = 1), *S. terrae* (*n* = 1), and *S. tumulicola* (*n* = 1).

A total of 2,213 genomes were ultimately included in this analysis. In this study, the genomes of unclassified species were all included as *Stenotrophomonas* sp. (including uncultured organisms). In addition to *S.* sp., the study encompasses 29 defined species composed of 1,841 genomes. The number of each species is shown in Figure 1A. *S. maltophilia* is the most abundant, with a total of 1,555 genomes, accounting for 70.27% of all genomes. The second most abundant species is *S.* sp., with a total of 372 genomes. The remaining species all have less than 50 genomes, including 12 species with only one genome. Genomic characterization of different species was shown in Figures 1B,C. The size range of all *Stenotrophomonas* genomes is from 1.77 to 6.98 Mb, while that of *S. maltophilia* is 3.80 to 5.54 Mb. The average genome size of each species is similar, except for *S. koreensis*, whose genomes are significantly smaller than other species, ranging from 2.07 to 3.09 Mb. *S*. sp. contains various unclassified strains, so its relatively wide genome size range is expected. but the performance of *S. nitritireducens* is beyond expectation. S. nitritireducens only has 6 genomes, but there is one largest (GCA_037190265.1, 6.98Mb) and one smallest (GCA_017304365.1, 1.92Mb) genome of genus Stenotrophomonas, although the average size of the other 4 genomes (4.22 Mb) is similar to other species.

The GC content of *Stenotrophomonas* genomes ranges from 62.54 to 70.17%. Apart from *S. maltophilia*, *S.* sp., and *S. nitritireducens*, the GC content distribution of each species is relatively concentrated (fluctuations do not exceed 2%). Although the GC content of over 95% of *S. maltophilia* genomes concentrates at 65.5–67.0%, it ranges from 63.6 to 68.17%. *S.* sp. strains are more dispersed, ranging from 62.54 to 69.87%. The GC content of *S. nitritireducens* genomes exhibited a bimodal distribution, with four genomes ranging from 68.08 to 68.62% and the other two genomes ranging from 63.83 to 63.98%. The GC content of *S. acidaminiphila* is higher than that of other species, ranging from 68.06 to 69.75%.

One reason for the large fluctuations in genome size and GC content is that 20.83% (461/2213) of the submitted genomes of *Stenotrophomonas* are metagenome-assembled genomes (Supplementary Table S2), and the majority of these outlier genomes belong to this type, limiting the accuracy of these genomes. The other speculated reason is that many isolates are from diverse sources, leading to genetic variability and diversity. There are nine strains that have been sequenced more than once and have genomes with different levels of completeness in the database. For example, *S. maltophilia* strain NCTC10498 has two complete genomes (GCA_011386925.1 and GCA_900475685.1) and one scaffold genome (GCA_014235265.1). The ANI values of genomes from different-levels of the same strain were similar to the reference genomes (Supplementary Table S6), indicating that the genome sequencing method and genomic assembly level do not affect the species classification based on the ANI.

### 3.2 Analysis of the reference genomes

**Figure 2:**
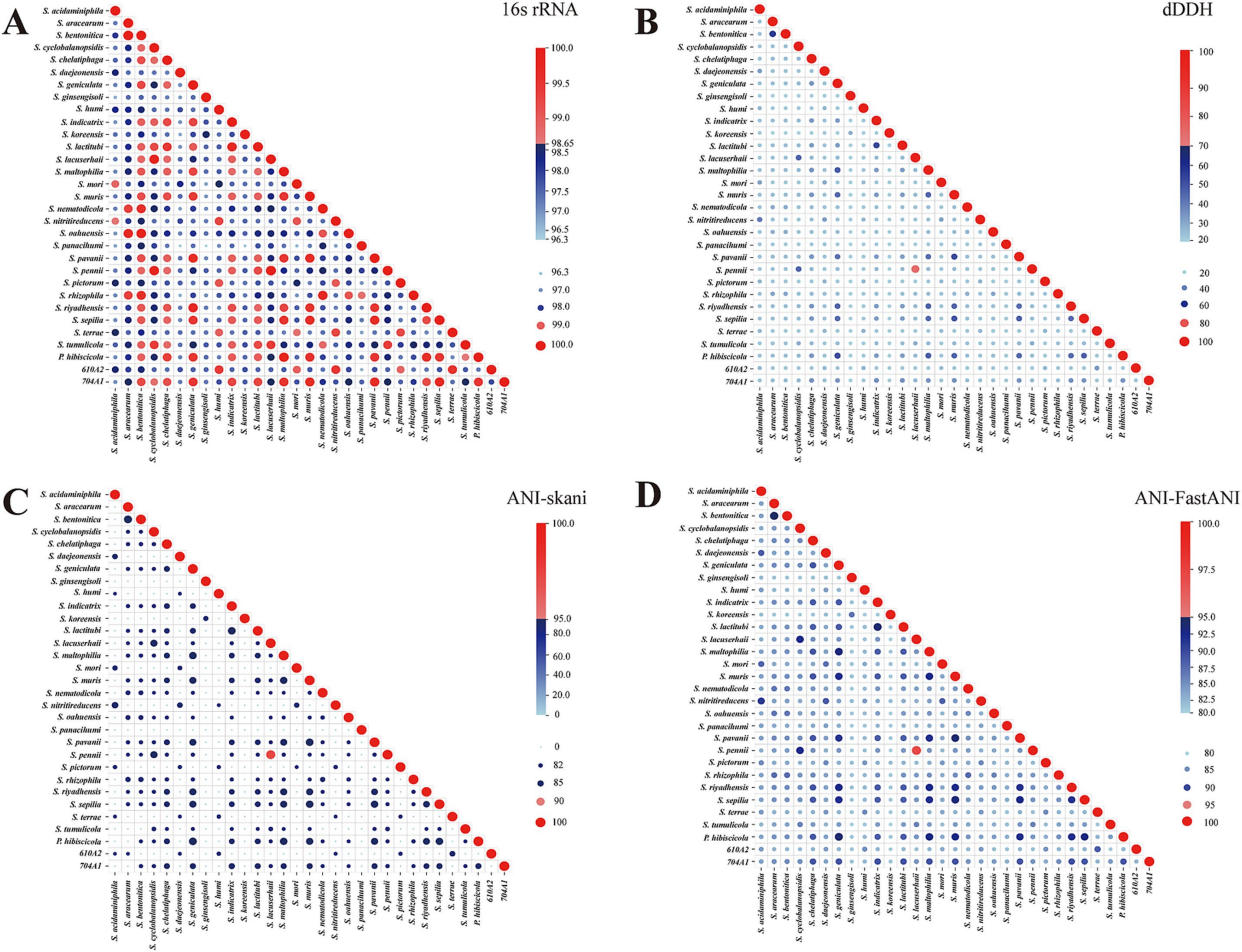
Pairwise 16S rRNA gene sequence identity, dDDH, and ANI of 31 reference genomes. **(A–D)**, respectively, represent 16S rRNA gene similarity, dDDH, and ANI calculated using skani and FastANI. They are all divided into two colors based on the species threshold (16S rRNA gene similarity 98.65%, dDDH 70%, and ANI 95%), with values greater than or equal to the threshold represented by shades of red from light to dark and values below the threshold represented by shades of blue from light to dark. The size of the bubbles indicates the magnitude of the values.

The 16S rRNA gene sequences were extracted, and pairwise 16S rRNA gene similarities were calculated (Figure 2A). The 16S rRNA gene similarity between many species is ≥98.65%, and even greater than 99%. Obviously, the 16S rRNA gene sequence has low discriminability for *Stenotrophomonas* species.

As the gold standard for the delineation of bacterial species, the trends of pairwise ANI and dDDH were essentially consistent across the reference genomes analyzed (Figures 2B–D). The dDDH and ANI values between the defined species are all below the commonly used cutoff values for species determination. The only exceptions were *S. pennii* Sa5BUN4 and *S. lacuserhaii* K32. Their 16S rRNA gene sequence similarity, dDDH, and ANI values calculated using skani and FastANI between these two species were 100, 74.5, 97.32, and 97.22%, respectively, all of which exceeded the species cutoffs. Although *S. pennii* was first reported in 2021 (Gilroy et al., 2021), it was not validly published. *S. lacuserhaii* was reported in 2022 (Deng et al., 2022) and validly published under the International Code of Nomenclature of Prokaryotes (ICNP). *S. pennii* and *S. lacuserhaii* should be merged into one species.

In addition, we observed that the ANI values between *S. panacihumi* GSS15 and other genomes were low, with values calculated using skani being below 82% and values calculated using FastANI ranging between 81 and 84%. *S. panacihumi* is a non-validly described and poorly characterized species (Yi et al., 2010). It only has two assembled genomes. The study by Vinuesa et al. also demonstrated the inconsistency of *S. panacihumi* in the phylogeny of the genus *Stenotrophomonas* (Vinuesa et al., 2018). This could be the reason why the GTDB classified *S. panacihumi* into the new genus *Stenotrophomonas_A*. We also discovered the anomaly of *S. koreensis* and *S. ginsengisoli* in ANI comparisons, although both were validly described species (Yang et al., 2006; Kim et al., 2010). In addition, the paired ANI between *S. koreensis* DSM 17805 and *S. ginsengisoli* DSM 24757 was 86.76% (skani) and 86.92% (FastANI). However, their ANI with the other 29 reference genomes is very low, with values below 82% (skani) or ranging between 81 and 82% (FastANI).

**Figure 3:**
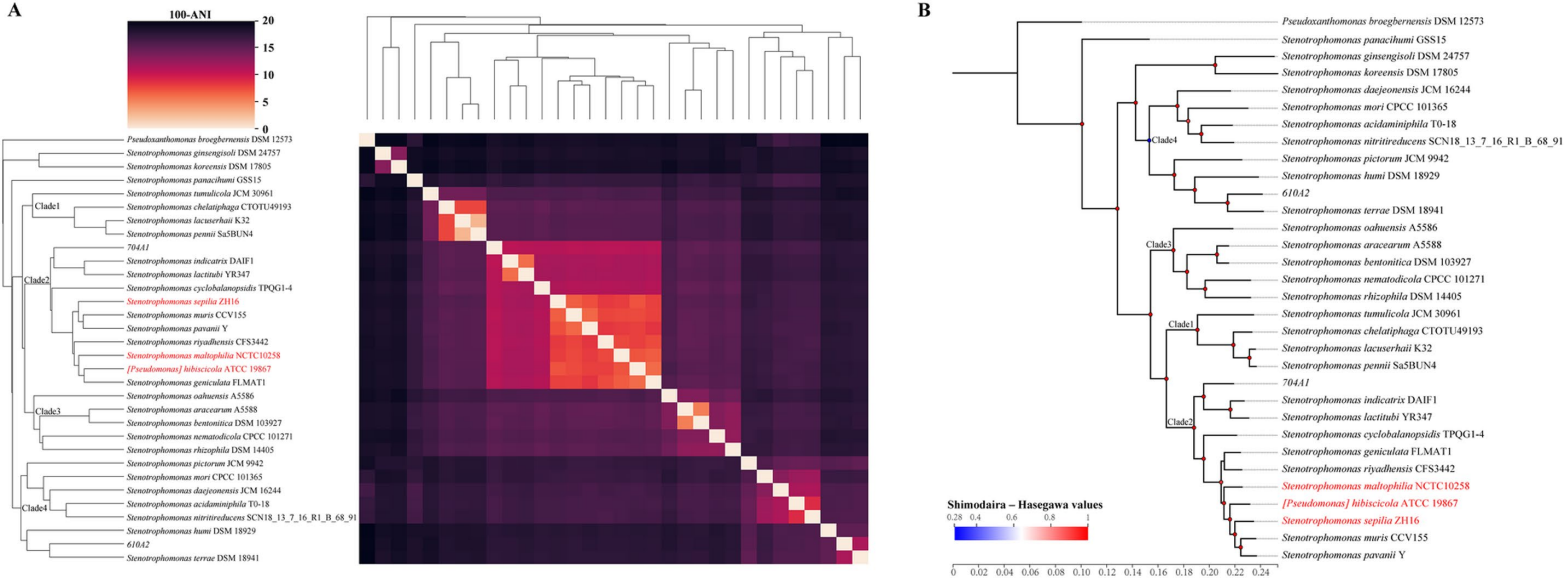
ANI clustered heatmap and phylogenetic tree of 31 reference genomes. **(A)** Clustered heatmap, based on the pairwise FastANI matrix of the 31 *Stenotrophomonas* reference genomes with *Pseudoxanthomonas broegbernesis* DSM 12573 as the outgroup, using average linkage hierarchical clustering. The shade of color represents the value of 100 – ANI; the closer the color is to black, the lower the ANI value is. **(B)** Core-genome phylogenetic tree: the values of the Shimodaira–Hasegawa test are shown by the gradient color circle. The red font indicates the Smc species currently listed in the NCBI database.

Furthermore, we compared the concordance between the ANI clustering heatmap (Figure 3A) and the phylogenetic tree constructed based on the core genes (Figure 3B). Considering that many pairwise ANI values between reference genomes are below 82% and output as “0” in skani as the default parameter setting (Shaw and Yu, 2023), we utilized FastANI to calculate accurate ANI values for clustering. The results showed that the classification of the four clades of the genus *Stenotrophomonas* based on these two methods and the composition of the species in each clade were similar. The relatively simple ANI calculation can replace the complex core gene analysis for identifying closely related species and classifying them on the phylogenetic tree. In the phylogenetic tree, we also observed inconsistencies between *S. panacihumi* GSS15 and other genomes of the genus *Stenotrophomonas*. While *S. koreensis* DSM 17805 and *S. ginsengisoli* DSM 24757 had lower ANI values than other genomes, they still belonged to the genus *Stenotrophomonas* on the phylogenetic tree.

### 3.3 Consistency between skani and FastANI

We compared the ANI values between all 1,841 genomes of 29 defined species and their reference genomes using two ANI tools to assess their consistency. The results indicated that when using 90 and 95 as thresholds, the ANI of each genome calculated by these two ANI tools was completely consistent in classification (Table 1 and Supplementary Table S3). The McNemar–Bowker test showed no significant difference (kappa coefficient = 1).

**Table 1:** Consistency between ANI calculated using skani and FastANI.

Skani	FastANI			Total
	ANI ≥ 95%	95%>ANI ≥ 90%	ANI<90%	
ANI ≥ 95%	1,083	0	0	1,083
95%>ANI ≥ 90%	0	600	0	600
ANI<90%	0	0	158	158
Total	1,083	600	158	1841

**Figure 4:**
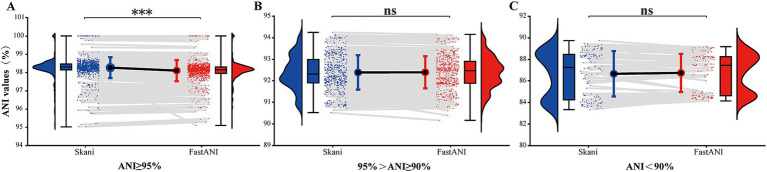
Raincloud plots of paired ANI comparisons calculated by two ANI tools. **(A–C)**, respectively, represent 1,083 ANI values greater than 95%, 600 ANI values between 90 and 95%, and 152 ANI values below 90% (excluding 6 pairs of values for which the skani output was 0). The plots were created using raincloud plots v0.2.0 (Allen et al., 2019) in R 4.4.0. Blue represents skani, and red represents FastANI. “***” indicates *p* < 0.001, “ns” indicates *p* > 0.05.

We conducted paired comparisons for ANI values across each range (Figure 4) and observed that for ANI ≥ 95%, ANI values calculated using skani were higher than those calculated using FastANI, exhibiting a notable statistical difference (*p* < 0.05). For ANI values between 90 and 95%, the ANI values computed by both tools were essentially consistent (*p* > 0.05). For ANI < 90%, the ANI values calculated using skani were slightly lower than those using FastANI. However, the difference was not statistically significant (*p* > 0.05). Despite a few differences in the ANI values calculated by the two ANI tools, they demonstrated identical discriminatory capabilities at thresholds of 90% or 95%. Considering that skani’s computations were several to tens of times faster than FastANI, we primarily utilized skani for large-scale paired ANI calculations in this study.

### 3.4 ANI clustering and evaluation of the ANI cutoff values for *Stenotrophomonas*

**Figure 5:**
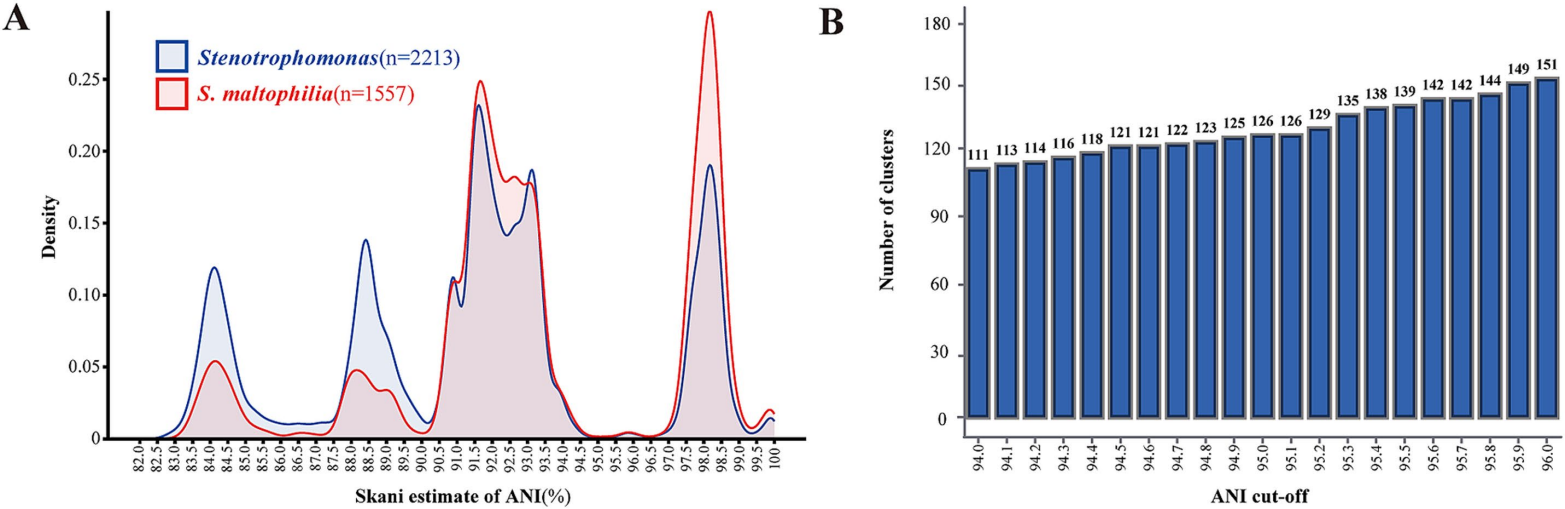
Clustering and distribution of ANI values. **(A)** Cluster heatmap of pairwise ANI comparison calculated for 2,213 *Stenotrophomonas* genomes, with colors ranging from light to dark, indicating nucleotide identity from high to low. **(B)** Kernel density plots of the distribution of paired ANI values of all *Stenotrophomonas* genomes and genomes annotated as *S. maltophilia*. **(C)** Clustering of 2,213 genomes based on pairwise comparison of ANI dissimilarity. The x-axis shows the ANI cutoffs from 94.0 to 96.0%, and the y-axis is the number of ANI clusters generated based on the ANI cutoffs.

We conducted paired ANI comparisons of all 2,213 *Stenotrophomonas* genomes and performed clustering. Out of a total of 4,897,369 comparisons of the genus *Stenotrophomonas* genomes, 4,196,423 yielded ANI values greater than 82%. For *S. maltophilia*, out of a total of 2,418,025 comparisons, 2,403,945 yielded ANI values in the 82–100% range. The distribution of paired ANI values of the genus *Stenotrophomonas* and *S. maltophilia* species is quite similar to the multimodal distribution (Figure 5A), which may be partially due to the fact that the genomes annotated as *S. maltophilia* account for the majority of the genus *Stenotrophomonas*.

We found that the peak of the ANI distribution is most abundant between 90 and 94.7%, accounting for approximately half of all ANI values. This indicated that numerous genomes have been mistakenly annotated as *S. maltophilia*, which actually belonged to other species closely related to *S. maltophilia* in the ANI clustering. The ANI values between the species classified in Smc, *S. sepilia*, *P. hibiscicola,* and *S. maltophilia* also fell in this range. Therefore, we recommended that a 90% ANI value could potentially serve as the threshold for distinguishing complex/group species within the genus *Stenotrophomonas*.

In our study, despite a minor peak between 95.5 and 96.5%, the curve representing ANI values between 94.7 and 96.5% remained flat and very low, not exceeding 0.45% of all pairs, especially around 95%. Therefore, we suggested that the interspecies ANI threshold for the genus *Stenotrophomonas* might fall within this range. We examined the number of ANI clusters formed at different ANI cutoffs from 94 to 96% with every 0.1% increment and did not observe a stable plateau in the number of clusters (Figure 5B). Considering that 95% is the nadir of the kernel density distribution curve, as the cutoff for species delineation in prokaryotes, ANI ≥95% can correctly classify the genus *Stenotrophomonas* at the species level.

### 3.5 Correct classification based on the ANI cutoff

In the ANI classification, 758 (41.17% of 1,841) genomes had ANI values below 95%, with 158 genomes (8.58%) even below 90% (Table 1). Table 2 provides a detailed list of species with ANI values below 95% compared to the reference genomes. These results indicated that a large proportion of genomes had been incorrectly identified and annotated. Meanwhile, only 135 out of 372 *S.* sp. strains had ANI > 95% compared to the reference genomes and could be classified into the defined species.

**Table 2:** Species inconsistent with the reference genome.

Species name	ANI ≥ 95%	95%>ANI ≥ 90%	ANI<90%	Total
*S. acidaminiphila*	25	17	0	42
*S. bentonitica*	2	0	10	12
*S. maltophilia*	850	583	122	1,555
*S. nitritireducens*	4	0	2	6
*S. rhizophila*	8	0	24	32
Total	889	600	158	1,647

Gröschel et al. classified Smc into 23 monophyletic lineages based on MALDI-TOF MS and 98.8% 16S rRNA gene sequence identity as the strain inclusion criteria (Gröschel et al., 2020). For consistency, we used and amended the naming convention of lineages from previous reports. In our study, 850 genomes, including the *S. maltophilia* reference genome NCTC10258, belonged to the lineage Sm6, which is strictly defined as *S. maltophilia* species by 95% ANI. Through ANI comparison, we discovered that the ANI values of several Smc lineages described by Gröschel et al. with species within or outside Smc were ≥ 95%, for example, Sm9 and Sm16, Sm3 and *S. sepilia*, and Sm5 and *P. hibiscicola* within Smc; Sm2 and *S. pavanii*, Sm4a and *S. muris*, Sm10 and *S. geniculata*, Sm15, and *S. riyadhensis*, and Sgin3 and *S. indicatrix* outside Smc. Moreover, it is worth mentioning that when the ANI threshold for the complex/group is set to 90%, only lineages Sm1–Sm18 should be kept in Smc, while lineages Sgn1-Sgn4 with more distant relationships should be removed.

The clustering heatmap of paired ANI comparisons of all 2,213 *Stenotrophomonas* genomes indicated that there are a considerable number of clusters within the genus *Stenotrophomonas* (Supplementary Figure S1). Using 95 and 90% ANI as species and complex/group thresholds, 2,213 *Stenotrophomonas* genomes in the NCBI database were re-clustered into 126 ANI genomic species and 51 ANI genomic complex groups (Supplementary Tables S4, S5). The clustering result in our study suggested that Smc should be redefined. It may consist of 1,668 genomes from 33 species, including 18 previously described species and lineages (*P. hibiscicola*/Sm5*, S. geniculate*/Sm10*, S. maltophilia*/Sm6*, S. muris*/Sm4a*, S. pavanii*/Sm2*, S. riyadhensis*/Sm15*, S. sepilia*/Sm3, Sm1, Sm4b, Sm7-8, Sm9(Sm16), Sm11-14, and Sm17-18) and 15 novel ANI species.

**Figure 6:**
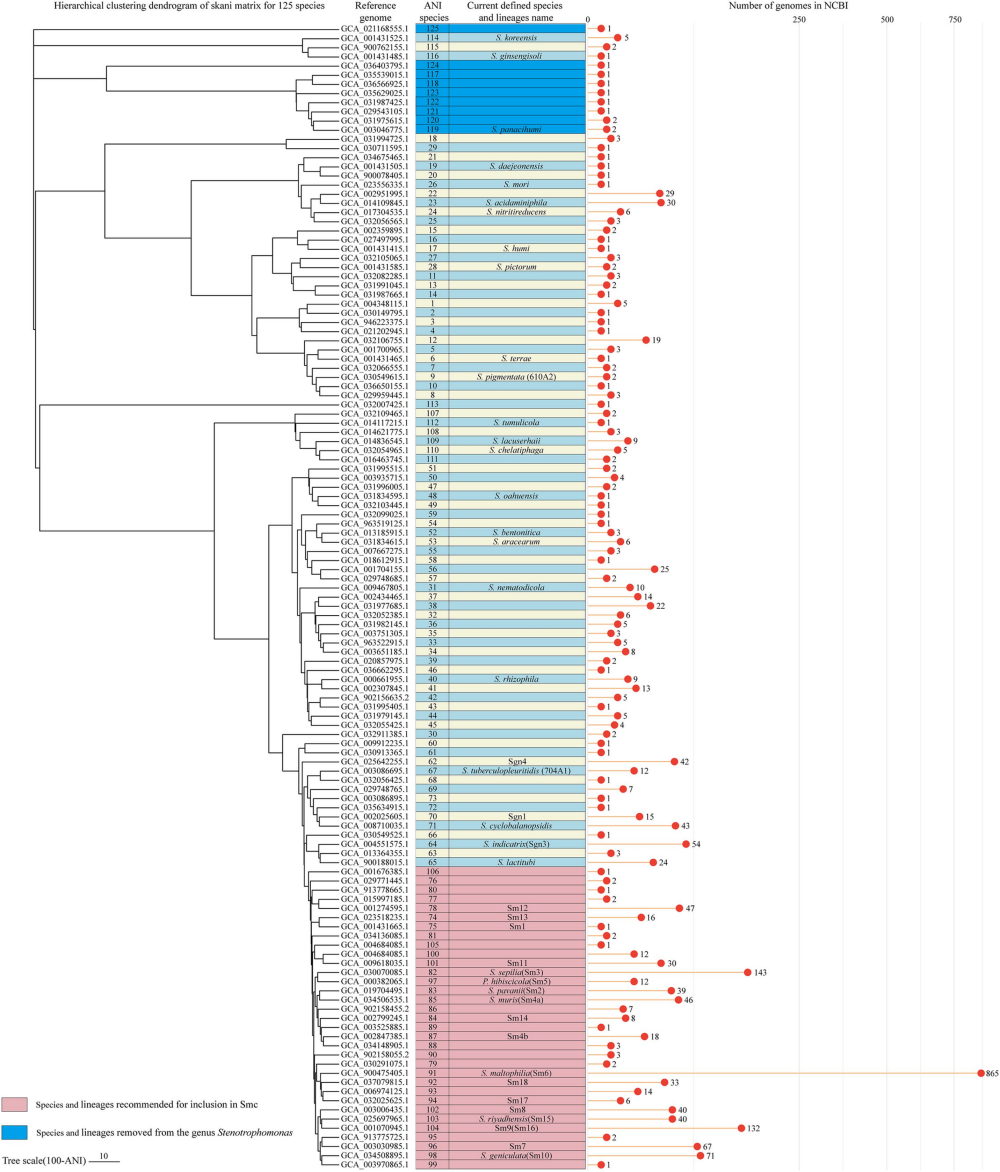
Hierarchical clustering dendrogram based on the skani matrix for the reference genomes of the 125 ANI genomic species. Average linkage hierarchical clustering was performed on the ANI matrix using Scipy v1.13.1, and the resulting Newick file was visualized in tvBOT. The first column indicates the corresponding ANI genomic species of each reference genome. The second column indicates the currently defined species and lineage names for these genomes. The lollipop plot shows the number of genomes belonging to each ANI genomic species (2,213 genomes in total).

The 10 genomes closely related to *S. panacihumi* were clustered into 4 complexes composed of *S. panacihumi* and other 7 ANI species (ANI species 117, 119–124). The ANI values between these genomes and other species in the genus *Stenotrophomonas* were below 82%. Whether plotted on the ANI clustering tree or the core gene phylogenetic tree, *S. panacihumi* is a clade that is far apart from other *Stenotrophomonas* species. Moreover, *Stenotrophomonas* sp. MMGLT7 (ANI species 125) should also be removed from the genus *Stenotrophomonas* as its genome (GCA_021168555.1) showed the highest identity with *Xanthomonas massiliensis* SN8 (GCA_900018785.1), with an ANI of 86.1%. The classification of *S. koreensis* and *S. ginsengisoli* was challenging. Their ANI values for other *Stenotrophomonas* species were even lower than those of *S. panacihumi.* However, on the core gene phylogenetic tree (Figure 3B), they were related to the clade including *S. terrae*, *S. nitritireducens,* and *S. acidaminiphila*. If *S. koreensis* and *S. ginsengisoli* are retained, there would be 116 ANI genomic species and 47 ANI complexes in the genus *Stenotrophomonas.* In addition to the aforementioned species and their reference genomes for other ANI species or lineages, high-quality genomes were selected to serve as their respective reference genomes. A hierarchical clustering dendrogram was constructed using the ANI matrix of 125 reference genomes and annotated with both ANI genomic species and currently defined species (or lineages) information (Figure 6).

It should be pointed out that ANI clustering is based on all genomes within a cluster, which may result in some ‘transboundary’ occurrences. For example, *S. lactitubi* Bi35 (GCA_918698175.1) exhibited ANI values above 95% when compared to the reference genome of *S. lactitubi* (GCA_900188015.1) and 10 other genomes within this species. However, its ANI values to the remaining 13 genomes of the species were < 95%. According to the genomic similarity to the reference genome, we kept it in *S. lactitubi*. In the 54 paired comparisons between lineage sm4b (18 genomes) and ANI species 88 (3 genomes), 12 values were > 95%, and 42 values were < 95%; therefore, they were divided into separate species.

## 4 Discussion

The genus *Stenotrophomonas* has been widely studied as its members have both clinical and environmental significance (Ryan et al., 2009; An and Berg, 2018; Pérez-Pérez et al., 2020; Kumar et al., 2023). Currently, species definitions of the genus *Stenotrophomonas* are based on a variety of methods including traditional physiological and chemotaxonomic properties, 16S rRNA gene, or genome sequence. In recent decades, bacterial taxonomic techniques and standards have undergone a series of changes, shifting from phenotypes that are easily influenced by the environment to stable single/multi-gene sequence analysis with higher resolution and further to the whole genome level. *Stenotrophomonas* is a genus with significant phenotypic and genomic variability intra-and inter-species. In this study, we proved that the 16S rRNA gene identity of many species within the genus *Stenotrophomonas* exceeded 98.65%, and the taxonomy based on the 16S rRNA gene was not effective and reliable in distinguishing *Stenotrophomonas* species.

The ANI has emerged as a rapid classifier of bacterial species, with a generally accepted 95% cutoff for a separate species (Wu et al., 2024). We assessed the consistency of the ANI and dDDH among the reference genomes of each species and found that they exhibited a consistent trend. Phylogenetic analysis based on core-genome alignments of reference genomes and ANI clustering were similar, especially in the identification of closely related species. We compared the consistency of two rapid ANI tools, skani (Shaw and Yu, 2023) and FastANI (Jain et al., 2018), in paired ANI values between *Stenotrophomonas* reference genomes and strains of their species. We discovered that when ANI values were > 95%, ANI values calculated using skani were slightly higher than those from FastANI, which has not been previously reported. This could be due to skani’s key operating regimes being for medium-to-high ANI (>82%) (Shaw and Yu, 2023). Apart from that, the consistency between skani and FastANI is very high. Considering the speed, convenience, and feasibility of large-scale analysis, we used the ANI value reported by skani for species classification.

The distribution of paired ANI values in the genus *Stenotrophomonas* showed a multimodal distribution, which significantly differed from the previously reported wide gap or lack of a gap between 83 and 95% typically observed in prokaryotes (Jain et al., 2018). This valley range suggests genetic discontinuity in prokaryotic genomes, a pattern that appears to be disrupted in the genus *Stenotrophomonas*. This reflects that the genus *Stenotrophomonas* is genetically and phenotypically heterogeneous (Ryan et al., 2009; Vasileuskaya-Schulz et al., 2011; Berg and Martinez, 2015). The genus *Stenotrophomonas* has been shown to possess a number of functional properties, including, but not limited to, plant growth-promoting activity, nitrogen fixation, and rhizoremediation (Kumar et al., 2023). This diversity may be related to their genetic heterogeneity, which may be attributed to horizontal gene transfer. Zhao et al. demonstrated that *Stenotrophomonas* can introduce alien genes by exchanging genetic material with other community members in the habitat, promoting their broader genomic plasticity (Zhao et al., 2024).

We evaluated the ANI thresholds for the genus *Stenotrophomonas*, setting 90 and 95% as the thresholds for complex groups and species, respectively. Although the distribution of ANI values ranging from 94.7 to 96.5% was very low, we did not find a more suitable ANI cutoff value to replace the conventional ANI threshold of 95%. Based on the 95% ANI cutoff, we found many misclassified strains in the genus *Stenotrophomonas*, especially in *S. maltophilia* (*n* = 705, 45.34% of 1,555 strains). This may be the reason why Ciufo et al. chose 88.5% as the cutoff value for *S. maltophilia* (Ciufo et al., 2018). Similarly, this may explain why the genome size and GC content of *S. maltophilia* in our study are broader than those described in previous research (Xu et al., 2023).

Some species of this genus are pathogenic to humans. *S. maltophilia* is an important cause of hospital-acquired drug-resistant infections and is listed by the World Health Organization as one of the leading drug-resistant nosocomial pathogens worldwide (Brooke, 2012; Chang et al., 2015; Brooke, 2021). Lineage Sm6 (strictly defined as *S. maltophilia*) strains are potentially well-adapted to colonize or infect humans (Gröschel et al., 2020). Unfortunately, a considerable proportion of current *S. maltophilia* strains do not belong to *S. maltophilia*, but rather to other species within the Smc or even more distantly related species. Therefore, the pathogenicity and clinical significance of *S. maltophilia* and other species within the genus *Stenotrophomonas* need to be carefully considered. In other words, some infections caused by other species have been mistakenly identified as *S. maltophilia* infections, for example, Sm2 and *S. pavanii* (same species, ANI ≥ 95%). The *S. pavanii* strain obtained from sugarcane is widely used in organic agriculture (Ramos et al., 2011) to improve the yield and quality of many crops. According to the sample details of the genome from NCBI, only a small proportion (9/27) of *S. pavanii* was isolated from humans. However, 39 of 49 Sm2 strains were isolated from humans (Gröschel et al., 2020).

Furthermore, we observed anomalies in the phylogenetic tree of the core genes and ANI clustering of *S. panacihumi*, which is consistent with other studies (Vinuesa et al., 2018). Although *S. koreensis* and *S. ginsengisoli* had significantly lower ANI values, they still belonged to the genus *Stenotrophomonas* on the phylogenetic tree. Therefore, like the GTDB, we also recommend removing the whole *S. panacihumi* clade from the genus *Stenotrophomonas*, but the relationship of *S. koreensis* and *S. ginsengisoli* with the genus requires further biochemical and physiological studies for verification.

In conclusion, this study identified significant misclassified taxa within the genus *Stenotrophomonas* by ANI analysis. We identified an ANI cutoff of 95% for a genomic species delineation and 90% for genomic complexes/groups. After removing outliers, we defined 116 ANI genomic species and 47 ANI genomic complexes. Due to the opportunistic pathogenicity of some species of this genus, a well-defined ANI genomic species classification for *Stenotrophomonas* and Smc is crucial for species identification, diagnosis, clinical treatment, and epidemiological studies.

## Data Availability

The datasets presented in this study can be found in online repositories. The names of the repository/repositories and accession number(s) can be found in the article/Supplementary material.
