# Supplementary figures and images for: Revised taxonomic classification of the Stenotrophomonas genomes, providing new insights into the genus Stenotrophomonas
Source: Front Microbiol. 2024 Dec 12;15:1488674. doi: 10.3389/fmicb.2024.1488674 (PMC11669713; doi:10.3389/fmicb.2024.1488674)

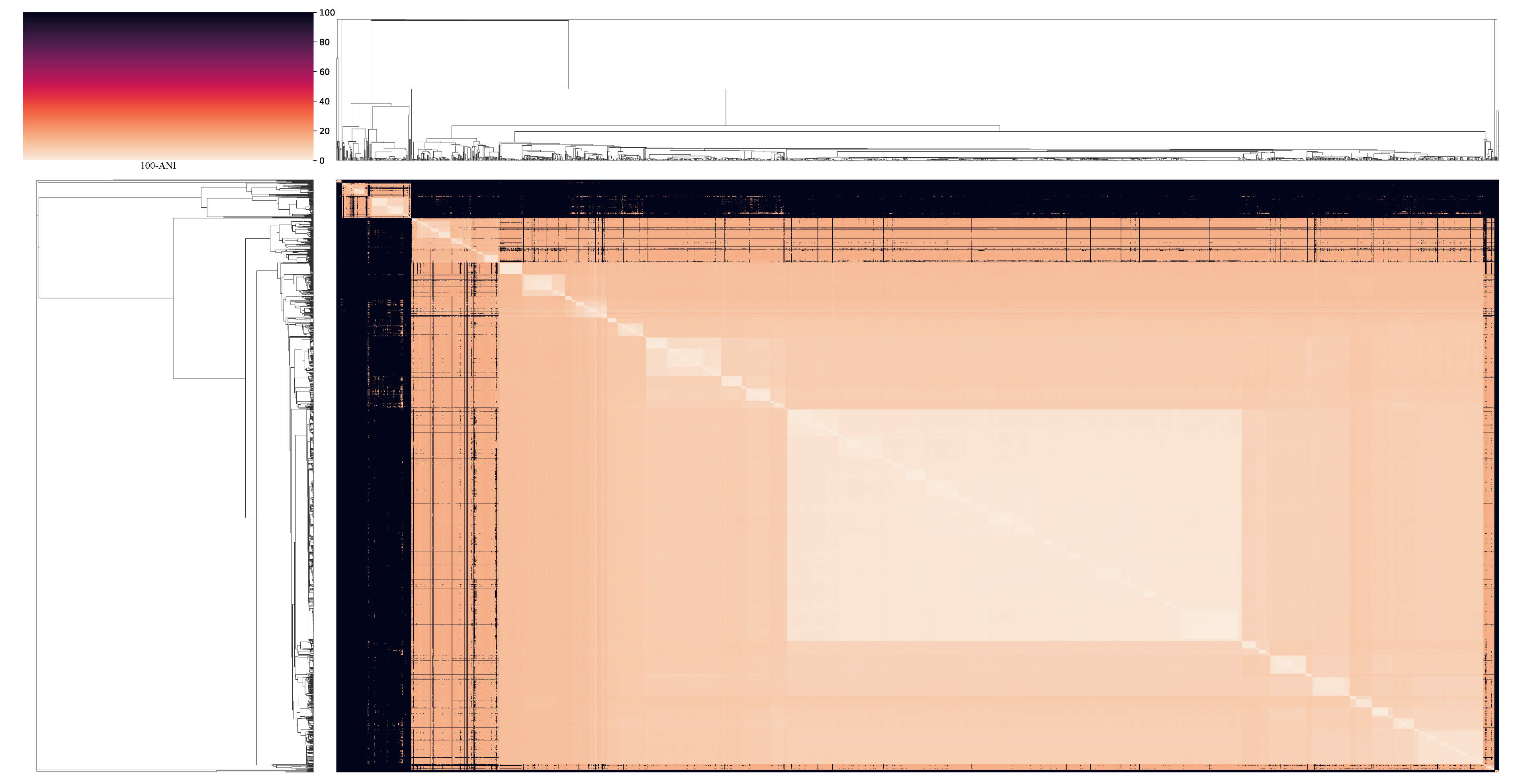

Supplement: Supplementary file 2 [file Image_1.TIFF]
